# Opinion: Do not treat

**DOI:** 10.1590/S1677-5538.IBJU.2016.02.04

**Published:** 2016

**Authors:** Wesley W. Ludwig, Justin B. Ziemba, Brian R. Matlaga

**Affiliations:** 1 James Buchanan Brady Urological Institute, Johns Hopkins Medical Institutions, Baltimore, MD, USA;; 2 Division of Urology, Department of Surgery, Perelman School of Medicine at the University of Pennsylvania, Philadelphia, PA, USA

**Keywords:** Urinary Calculi, Kidney Calculi, Hydronephrosis

The increasing use of cross-sectional imaging has made the incidental detection of asymptomatic stones a common occurrence with an estimated 8% of the population affected (1). Despite its frequency the average asymptomatic stone size is only 3 mm and is frequently located in the lower pole (1, 2). Nevertheless, urologists are often faced with the decision to treat or not to treat these asymptomatic stones. Favoring treatment is the theoretical benefit of preventing a future symptomatic stone event. Against treatment is the potential for morbidity related to our treatment modalities in the setting of a stone which may never become symptomatic. Therefore, we argue that avoidance of treatment in an asymptomatic lower pole renal stone < 1 cm, especially initially, in favor of surveillance is the best management choice.

The natural history of untreated lower pole renal stones has been previously described in mostly single-institution, retrospective case series. Despite this limitation these investigations provide the best evidence as to the symptomatic progression of these stones. In the largest study to date, Burgher et al. followed 300 patients for over 3 years with asymptomatic stones, half of which were located in the lower pole (mean size 11 mm). In the lower pole cohort, 61% experienced stone growth, 50% experienced pain, and only about 25% required intervention (3). In a more contemporary study, which followed 110 patients for over 3 years with asymptomatic renal stones (mean size 7 mm), only 24% became symptomatic with 19% requiring surgical intervention. However, 19% increased in size and the spontaneous passage rate was a diminutive 2.9% (2). In a smaller study of 24 patients, but who were observed for the longest time over 4 years, demonstrated similar results. . Only 11% of patients underwent surgery and not a single patient required intervention within the first two years of follow-up, suggesting that early observation is very reasonable (4). A study of 50 patients with stones (mean size 5.7 mm), around half of which were in the lower pole, followed over a 4 year period, reported a significant spontaneous passage rate of 20% and only 7% required intervention (5).

Taken together these studies suggest that the natural history of asymptomatic lower pole renal stones rarely require intervention, although they do have a slightly higher rate of symptomatic events and growth over the intermediate term. Given the overall stability of these stones we advocate for initial observation with treatment reserved for after a symptomatic event.

While the observation of asymptomatic lower pole stones appears to involve minimal risk, a number of randomized trials have compared the outcomes of surveillance, ureteroscopy (URS) and extracorporeal shock wave lithotripsy (ESWL). In assessing the value of these modalities, it is important to take into consideration the complications of these procedures in an otherwise asymptomatic patient. In a trial that randomized patients with lower pole stones less than 1 cm to ESWL or URS, stone free rates were 35% and 50%, respectively. However, 20% of patients undergoing URS experienced intraoperative complications with a similar percentage of patients experiencing post-operative complications in both URS and ESWL (6). Therefore, by proceeding to immediate treatment there is always the risk of patient morbidity.

ESWL is commonly utilized for treatment of asymptomatic lower pole calculi as it is non-invasive and well tolerated. A randomized trial compared ESWL to observation for asymptomatic renal stones with nearly 2/3 of the stones located in the lower pole. No significant differences were observed for stone free rate (28% v 17%), need for additional treatment, symptoms, or quality of life (7). A recent study by Sener et al. randomized patients with asymptomatic lower pole stones less than 1 cm to URS, ESWL, or observation. In the treatment groups, the stone free rates following URS and ESWL were excellent (90% v 92%). In the observation group, only 12% of patients became symptomatic, which was defined as pain, obstruction, or infection. Interestingly, this was the same rate of complications observed in the treatment group. For those who underwent URS, 14% experienced a complication with 6% considered major (Clavien grade III-V) and for those who underwent ESWL, 6% experienced a complication (8). In a trial that included patients with a larger stone burden, but still less than 2 cm, randomized patients to percutaneous nephrolithotomy (PCNL), ESWL, or observation. The rate of intervention in the observation group was only 19% with no adverse outcomes while delaying treatment. All patients received post-intervention imaging to document renal scar. In the treatment groups, 3.2% and 16% of patients developed a renal scar in the PCNL and ESWL groups, respectively. Not surprisingly not a single patient in the observation group developed a renal scar (9).

These randomized studies suggest that treatment results in an excellent stone free rate, but that the benefit to the patient is modest at best given that observation resulted in equivalent symptomatic outcomes. Furthermore, with any treatment there is always the risk that a patient who is asymptomatic at the time of diagnosis then suffers a complication, which can be morbid. Therefore, again, this suggests that delaying treatment is reasonable.

Although small lower pole stones have a low spontaneous passage rate, only about 25% will become symptomatic and require intervention in short to intermediate term follow up. Immediate treatment does not demonstrate improved outcomes, and with the risk of complications may be more of a risk than a benefit. Untreated renal stones certainly have the capability to increase in size and eventually lead to a symptomatic event. Therefore, periodic surveillance is required with attention to specific risk factors such as age, gender, and stone history which may suggest early progression and the need for closer follow-up (10). In fact, observation is a recommendation endorsed by the European Association of Urology for asymptomatic calyceal stones with treatment reserved for a symptomatic event (11). Therefore, we advocate for initial observation with a delay in treatment until there is an indication, which likely results in little risk to patients while preventing many an unnecessary invasive procedure.
